# Understanding Miscarriage Prevalence and Risk Factors: Insights from Women in Jordan

**DOI:** 10.3390/medicina60071044

**Published:** 2024-06-26

**Authors:** Zina Al-Alami, Rana Abu-Huwaij, Shereen Hamadneh, Esra’ Taybeh

**Affiliations:** 1 Department of Basic Medical Sciences, Faculty of Allied Medical Sciences, Al-Ahliyya Amman University, Amman P.O. Box 19328, Jordan; 2 College of Pharmacy, Amman Arab University, P.O. Box 2234, Amman 11953, Jordan; r.abuhuwaij@aau.edu.jo; 3 Department of Maternal and Child Health, Princess Salma Faculty of Nursing, Al al-Bayt University, Mafraq P.O. Box 130040, Jordan; shereenh@aabu.edu.jo; 4 Department of Applied Pharmaceutical Sciences and Clinical Pharmacy, Faculty of Pharmacy, Isra University, P.O. Box 22, Amman 11622, Jordan; esra.taybeh@iu.edu.jo

**Keywords:** fertility, Jordan, miscarriage, pregnancy risk factors, sustainable healthcare

## Abstract

*Background and Objectives*: Miscarriage is a complication that is influenced by many risk factors that have been reported in different studies and that vary among countries. Despite the influence of various known risk factors for miscarriage, 30% to 50% of miscarriages are from unidentified causes. The aim of this study is to determine the prevalence of miscarriages in Jordan and the associated risk factors. *Materials and Methods*: A cross-sectional online survey was conducted in Jordan among married women to investigate the prevalence of miscarriages and identify potential risk factors. *Results*: Women (*n* = 704) were surveyed, and 17.9% reported a history of miscarriage. The identified risk factors were being an active smoker during pregnancy, having more than four children, having a family history of miscarriage, having fertility problems, receiving medical assistance for conception, and traveling by air during pregnancy. *Conclusions*: The results suggest that there are both modifiable and non-modifiable risk factors for miscarriages in Jordan and that a proportion of these may be preventable. The findings can be used to enhance patient awareness and inform policy development to decrease the incidence of miscarriage in the country.

## 1. Introduction

Miscarriage, also known as pregnancy loss or spontaneous abortion, is the term most frequently used to describe a nonviable intrauterine pregnancy up to 20 weeks of gestation. Miscarriage is a complication of pregnancy that globally affects 12–15% of pregnant females [1].

Many investigations have been conducted to evaluate the prevalence of miscarriages worldwide [2,3]. A miscarriage is a multifactorial complication that is influenced by many risk factors that have been reported in different studies and vary among countries. Factors could include mothers who are young (age < 20) or older (age > 35), have had prior miscarriages, have very low or very high body mass indices, work night shifts, drink alcohol, and have been exposed to air pollution, stress, smoking, and pesticides [4].

Despite the influence of several known risk factors for miscarriage, 30% to 50% of miscarriages are from an unknown cause [5]. Moreover, the implications of miscarriages extend beyond the individual level, impacting women, their families, and society. By understanding the multifactorial nature of miscarriages and identifying both modifiable and non-modifiable risk factors, healthcare providers and policymakers can work towards reducing the incidence of miscarriages, improving reproductive health outcomes, and providing comprehensive support to those affected women [4].

The predictors of miscarriage in low- and middle-income countries have not been properly investigated. Until reliable information on the risk factors of miscarriage in such countries is compiled, it is challenging to construct and implement effective preventive interventional strategies. Consequently, the findings of this study can be utilized to enhance patient awareness regarding potential risk factors and preventive measures, and empower women with the knowledge about modifiable risk factors. Thus, the objective of this research was to determine the population-level prevalence of miscarriages in Jordan as well as the contribution of various prospective risk factors on miscarriage.

## 2. Material and Methods

### 2.1. Study Design

A cross-sectional study was undertaken employing an online open survey during April–July 2022 with voluntary participation for women in Jordan. Any married woman in Jordan was allowed to take part in this research, irrespective of current marital status (divorced, separated, or widowed) and miscarriage experience. To ensure statistical reliability, a minimum sample size of 385 responses was determined. This calculation was based on a 95% confidence level, a 5% margin of error, and a presumed response distribution of 50%, as computed using the Raosoft sample size calculator (http://www.raosoft.com/samplesize.html, accessed on 13 May 2022).

### 2.2. Study Tool

The questionnaire development process was defined, involving key stages. Initially, a comprehensive literature review ensured coverage of all relevant aspects. Validation enlisted three independent experts (a gynecologist, family medicine specialist, and nursing associate professor) whose input refined the questionnaire. A pilot study with 20 women informed further refinement, incorporating participant feedback into the final version, which was published online using Google Forms for data collection from April 2022 to August 2022.

The Cronbach alpha coefficient test was employed to assess reliability, yielding a 0.791 coefficient. This indicates robust internal consistency, affirming the questionnaire’s reliability in measuring women’s health variables.

The questionnaire encompassed 39 questions, categorized into three sections: sociodemographic data (8 questions), gynecologic and obstetric history (11 questions), and potential risk factors associated with miscarriages (20 questions).

The sociodemographic section sought information on various aspects, including the participant’s current marital status, age, height, body weight, nationality, place of residency, employment, monthly family income, and educational level, as well as smoking habits for both the participant and her husband.

The second section of the questionnaire focused on gathering information pertaining to gynecologic and obstetric history. The questions encompassed various aspects, including the age at which the first menstruation occurred, the duration and daily volume of menstrual bleeding, the length of the menstrual cycle, and the presence of any reproductive system disorders or conditions such as polycystic ovarian syndrome, fibroids, endometriosis, and others. Additionally, this section sought information regarding current or past issues related to infertility for both the participant and her husband, the number of children, whether the last pregnancy was achieved through natural conception or assisted reproductive methods, and whether there was a family history of miscarriages.

The final section focused on potential conflicting clinical, environmental, and lifestyle risk factors of miscarriage. Participants were asked about exposure to various conditions or situations during their last pregnancy, such as fever, infections, medications, radiation, accidents, or carrying heavy items. It also included questions about hair dyes and other chemicals, stress, anxiety and/or depression, chronic diseases, prior use of oral or intrauterine contraceptives, air travel, vaccines, special diet, caffeine consumption, vigorous exercises, transvaginal ultrasound, having negative attitudes toward the pregnancy, and whether there was a family relationship between the participant and her husband.

### 2.3. Data Collection

The data collection process involved distributing a link through social media. Participants were provided with all necessary information and objectives on the first page of the questionnaire. By responding “Yes” to the opening question, which asked if they willingly wished to take part in the research, respondents indicated their agreement. Those who chose not to participate had no obligation to complete the questionnaire.

The introduction of the questionnaire explicitly stated that no personal information would be requested, and all data would be treated with professionalism and confidentiality, adhering to ethical and scientific research standards. Participants were informed that they could withdraw their responses from the survey at any time without having to provide reasons. No incentives were offered as encouragement for completing the survey. This study received ethical approval from the Institutional Review Board (IRB) at Al-Ahliyya Amman University, with the approval number MM 1/4-2022, on the 24th of April 2022.

### 2.4. Data Analysis

The responses were exported to an Excel sheet and subsequently coded for analysis using SPSS version 26 (SPSS Inc., Chicago, IL, USA). Descriptive analysis was conducted, presenting frequencies and percentages. To compare differences between the miscarriage and no miscarriage groups, a chi-square test was employed, with a *p*-value < 0.05 being considered statistically significant. For variables with a *p*-value < 0.05, binary logistic analysis was applied to identify the contributing factors associated with miscarriage.

## 3. Results

Out of the 714 women who accessed the survey link, 704 women in total willingly agreed to participate and successfully completed the questionnaire. The utilization of an online questionnaire facilitated a larger sample size and improved the response rate, resulting in a collection of responses that exceeded our initial estimates. Table 1 displays the sociodemographic characteristics of the participants. Approximately 46.3% of the participants fell within the age range of 22 to 34 years old, 52.0% resided in North Jordan, and 48.7% reported an income of less than JOD 500. The most prevalent educational degree among the participants was a bachelor’s degree, accounting for 44.7%, and 45.7% of them were employed. Notably, most women identified as non-smokers (78.7%) while over half of the participants’ husbands (57.5%) were reported to be smokers.

Approximately 17.9% of respondents (*n* = 126) indicated a history of miscarriage. The prevalence of miscarriage did not significantly differ by demographics, except for educational level (*p* = 0.035) and smoking status (*p* = 0.028). Age, place of residency, employment status, income, and calculated body mass index were not found to be significantly related to a history of miscarriage.

Regarding age at menarche, around half of the participants (52.3%) experienced it between 11 and 13 years. Only a small percentage (5.3%) reported early menarche (<11 years). Most participants (84.9%) had a blood flow duration of 3–7 days during menstruation. The menstrual cycle length for about two-thirds of the participants fell within 21–35 days while 10.1% reported having irregular cycles.

**Figure 1:**
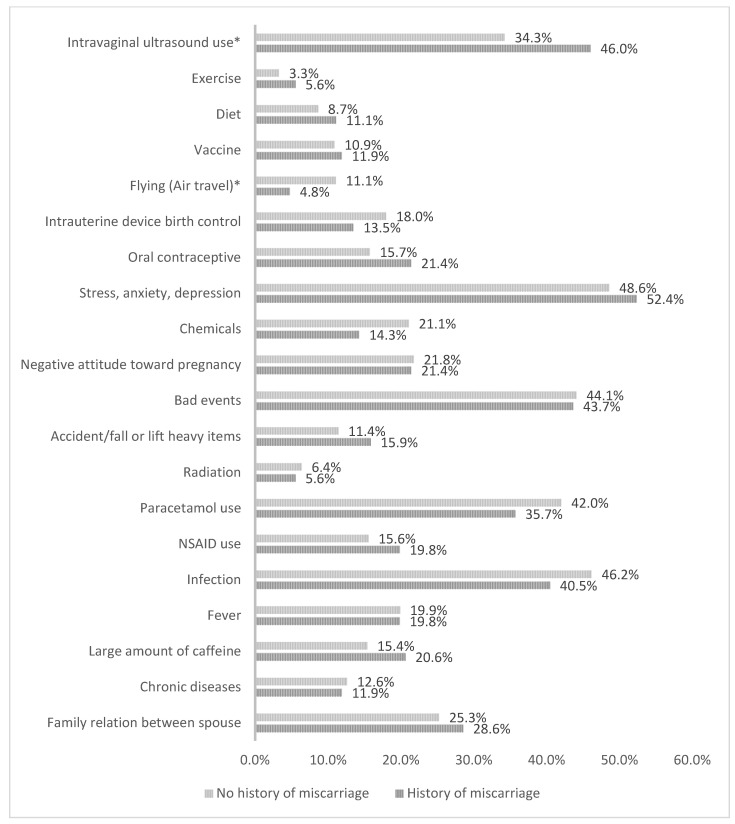
Potential risk factors of miscarriage (* *p*-value < 0.05). NSAID: non-steroidal anti-inflammatory drugs.

Figure 1 displays the potential risk factors of miscarriage during their most recent pregnancy. A considerable number of women reported exposure to potential risk factors for miscarriages. A considerable number of participants experienced stress, anxiety, depression, or adverse life events, such as the death of a close relative or friend. Approximately 52.4% of participating women who had experienced a miscarriage and 48.6% of those with no history of miscarriage reported symptoms of stress, anxiety, or depression. However, the self-reported presence of these psychological conditions did not demonstrate a significant link to causing miscarriage. The existing evidence on the association between psychological stress and miscarriage is conflicting [6]. While some scholars support the belief that psychological stress and life events during pregnancy may increase the risk of miscarriage [6,7], further investigation is essential to gain a clearer understanding of the potential impact of psychological factors on the occurrence of miscarriages.

Moreover, women who had a miscarriage in their last pregnancy reported higher exposure to transvaginal ultrasound checks (46.0%), compared with those with no history of miscarriage. However, the subsequent regression analysis revealed that this factor was no longer linked to miscarriage. Although most gynecologists and reproductive health providers ensure that transvaginal ultrasound is generally safe for both the patient and fetus with no risk of causing a miscarriage, it is important to note that some participants in the study expressed concerns or fears about transvaginal ultrasound causing miscarriage. Therefore, it is essential for healthcare providers to address and alleviate any fears or concerns patients may have regarding medical procedures, particularly since in a cross-sectional survey about the women’s preferences for preterm birth screening, a quarter of the participants reported discomfort and about one-tenth of the participants reported bleeding as negative concerns after transvaginal ultrasound [8].

Additionally, despite the high prevalence of paracetamol use throughout pregnancy that falls between 35.7% and 42.0%, it has not been associated with an increased risk of miscarriage similar to the reported range of 42.0% to 65.1% from previous research [9].

Table 2 displays the results of the logistic regression analysis, which identified several significant risk factors associated with miscarriage. Smoking participants, whether using cigarettes or shisha, had a higher likelihood of experiencing miscarriages in their pregnancies compared with non-smokers (odds ratio (OR) 1.78; 95% confidence interval [CI] 1.09–2.89).

Additionally, having a family history of miscarriage, having more than four children, and conceiving after medical assistance were also found to be significantly associated with miscarriage (*p* < 0.05). The number of children was also significantly associated with increased odds of reporting miscarriages (OR 2.51, 95% CI 1.23–5.12).

Furthermore, although a relatively small percentage of women reported experiencing gynecological disorders (13.8%) or fertility problems (17.5%) in themselves or their husbands (8.4%), such issues were significantly associated with miscarriage in the study (*p* < 0.05). Women with fertility problems and those who required medical assistance for conception had higher odds of experiencing miscarriages compared with those without fertility issues and those who conceived naturally (OR 2.48, 95% CI 1.44–4.27; OR 2.65, 95% CI 1.01–6.96, respectively).

Moreover, although a small number of women who lost their pregnancy had air travel experience (4.8%), a significant association was found between air travel and miscarriage. Pregnant women who had air travel experience during their pregnancy were 2.7 times more likely to experience miscarriage (CI 1.09–6.73) compared with those who did not travel by air.

## 4. Discussion

Miscarriage is one of the problems that happens during pregnancy in humans. Abortion, whether spontaneous (miscarriage) or induced, is defined as ‘‘the expulsion or extraction from its mother of a fetus or embryo weighing 500 g or less’’ [10]. It has been previously reported that the risk factors associated with miscarriage are consumption of caffeine, tobacco, alcohol, and drugs such as cocaine and heroin, together with previous miscarriages and induced abortion, maternal age, chromosomal abnormalities, uterine anatomic defects, menstrual, endocrine and immunological disorders, and some maternal infections [11]. Therefore, this current study aimed to determine the prevalence of miscarriages in Jordan and the associated risk factors.

The study examined miscarriages among married women in Jordan and explored the related risk factors during the period of April to August 2022, following the COVID-19 crisis. The estimated prevalence of miscarriage was 17.9%, which represents a noticeable increase when compared with previously estimated population rates. Globally, roughly 23 million miscarriages occur annually, resulting in an average of 44 pregnancies ending prematurely every minute. When examining all recognized pregnancies, the combined likelihood of miscarriage was 15.3%. In addition, the occurrence of two miscarriages was approximately 1.9%, with a range of 1.8% to 2.1%, while three or more miscarriages affected approximately 0.7% of women, with a range of 0.5% to 0.8% [4]. It is important to note that this significant variation in prevalence may be influenced by various factors, including individual lifestyle choices, demographics, and other variables that can elevate the risk of experiencing a miscarriage.

To better understand the reasons behind this higher prevalence, it became crucial to explore the potential impact of various variables on miscarriage rates. The study shed light on the risk factors most strongly associated with miscarriages in Jordan. Consistent with previous research, it was observed that maternal smoking was linked to an increased risk of early miscarriage, and there was a dose–effect relationship between smoking and this risk [12]. Moreover, a comprehensive systematic review and meta-analysis examining the relationship between smoking and miscarriage from 1956 to 2011 revealed that active smoking during pregnancy was associated with a higher risk of miscarriage, which escalated with the amount smoked. Additionally, exposure to second-hand smoke during pregnancy was found to elevate the risk of miscarriage by 11% [13]. Furthermore, smoking was related to recurrent pregnancy loss in a cohort study of 2829 Danish women, and smokers with recurrent pregnancy loss were reported to be younger in age than never-smokers [14]. Given the high prevalence rate of smoking in Jordan [15,16], it is crucial to implement tobacco control programs to reduce smoking rates and mitigate potential risks among pregnant women. Such initiatives would be essential to safeguard the health and well-being of expectant mothers and their unborn children in the country. In addition, in their commentary, Farioli and his research group documented that tobacco smoke exposure did not increase the miscarriage risk for women between 25 and 29 years, while active smoking increased the risk for other age groups. They related these finding with the age-related differences in metabolism [17].

Likewise, pregnancy gravida and parity are significant indicators that maternal healthcare providers must take into account and assess. In the current study, it was observed that miscarriages were more likely to occur among mothers with higher gravida and those who have a greater number of children. Pregnant women with four or more children had an approximately two and a half times higher chance of experiencing miscarriages compared with childless mothers. Similarly, a study conducted by Poorolajal and colleagues [5] found a direct association between miscarriage and parity, suggesting that the probability of miscarriage in nulliparous women (those with no previous pregnancies) is lower than that in primiparous (those with one previous pregnancy) or multiparous women (those with multiple previous pregnancies). Having more children increases social and care demands along with a potential impact on the economic status of the extended family. This situation significantly affects the risk of miscarriage. Economic status was reported to be a predictive factor for the risk of miscarriage [18,19,20]. These findings highlight the importance of considering both gravida and parity as valuable factors in understanding and addressing miscarriage risks in maternal healthcare.

Additionally, the findings revealed a significant association between miscarriages and the family history of mothers. Specifically, the presence of a family history of miscarriage was found to elevate the risk of experiencing a miscarriage. This is in line with the Woolner study, which concluded that women who miscarry may be more likely to have a family history of miscarriage. It is important to highlight that in this study, we fulfilled the recommendation from Woolner on performing more epidemiological studies with high-quality population data so as to support or contradict this relationship [21]. Not only family history but also the risk of miscarriage increases fourfold after three successive prior miscarriages [22].

Furthermore, the current study found that the likelihood of miscarriage among women with fertility problems and those who had conceived via medically assisted reproduction was higher than in those without fertility issues and those who had natural conception. On the contrary, in a large cohort study from 2019 on 10,011 women who conceived via medically assisted reproduction, none of the fertility treatment types were associated with an increased risk of miscarriage compared with that in spontaneous conception women [23]. Yang and his colleagues also found that neither primary nor secondary infertility among 15,210 pregnancies was associated with miscarriage [19]. Along with that, the analysis of Bu and his group demonstrated that early spontaneous miscarriages following assisted pregnancy were independently correlated with the mother’s age, previous number of miscarriages, or the endometrial thickness on the embryo transfer day. They also suggested that the risk of miscarriage might be higher if it is associated with other factors such as polycystic ovarian syndrome or malformations in the uterus along with other factors [24].

Moreover, most commercial airlines allow pregnant passengers to fly up to the 36th week of pregnancy because air travel is usually regarded as safe and does not appear to be hazardous to pregnancy [25]. However, in another study, there was a higher miscarriage rate among flight crew, and this higher rate was not related to passive smoking [26]. This study found that pregnant women who flew throughout their pregnancy had a 2.7-fold higher risk of miscarriage than those who did not. Air travel during pregnancy nowadays is very common, and the recommendation of the American College of Gynecology (ACOG) does not restrict air travel during pregnancy. Yet, more research is needed on the impact of travel-related factors (i.e., short-, medium-, or long-haul flights), as well as pregnancy-related factors (i.e., gestational week and miscarriage history) on the risk of miscarriage.

This nationwide design study provided extensive participant information, enabling the examination of various established maternal factors. However, the findings were limited by typical constraints associated with survey-based research. Another potential limitation was the predominantly retrospective self-report process used to identify potential risk factors for miscarriage. As a result, to facilitate international comparisons of miscarriage rates, address identified miscarriage risk factors, reduce recurrent miscarriages, and expedite research for informed policymaking, it is advisable to implement effective interventions and advocate for the comprehensive collection and reporting of miscarriage data across all healthcare clinics. Additionally, given that the data were collected in the aftermath of the COVID-19 crisis, it is plausible that the pandemic might have affected the health of pregnant mothers. Although much uncertainty remains, there could be a possible association between COVID-19 infection and miscarriage. Moreover, it is essential to emphasize the importance of capturing and analyzing miscarriage data across diverse populations and geographical locations to create a more comprehensive global perspective on this issue. By incorporating data from various regions and demographics, researchers and policymakers can gain valuable insights into the underlying causes and potential risk factors associated with miscarriage, leading to more targeted interventions and improved maternal healthcare outcomes.

By shedding light on the factors influencing miscarriages in such regions and the reported emotional impact of miscarriage on women [27], the study offers an opportunity to develop more effective preventive and intervention strategies that can be tailored to the specific challenges and resources of these countries. A portion of miscarriages could be preventable through behavior modifications, such as smoking during pregnancy and seeking medical assistance for conception. Effective intervention strategies can enable women to make informed decisions and take proactive steps to reduce their risk of experiencing a miscarriage.

Overall, the study’s contribution to understanding miscarriage predictors and preventable factors in Jordan lays the groundwork for future research in this field. By integrating these findings into public health strategies, healthcare professionals and policymakers can actively work towards reducing the burden of miscarriages in Jordan and potentially in similar contexts worldwide.

Focusing on the strengths of this current work, we suggest that healthcare authorities and researchers utilize these insights to design targeted public health campaigns and interventions aimed at mitigating preventable risk factors and improving reproductive health outcomes. In addition, we propose that data that can help identify all the risk factors behind miscarriage be collected by gathering information on demographics, health-related lifestyle and habits, and reproductive health. Moreover, engaging stakeholders is necessary to gathering feedback from women who have suffered from miscarriages, which could be achieved by community specialists of reproductive health, academics, researchers, and physicians working together. Moreover, it is our recommendation that campaigns and research-based public health initiatives be developed that designed specifically to identify miscarriage risk factors, and to spread culturally and linguistically suitable information through a variety of networks, including media. Another point is starting educational initiatives by distributing flyers and educational materials listing frequent miscarriage risk factors, such as smoking, alcoholism, and obesity, and providing advice on changing mothers’ lifestyles to minimize the risk factors, such as through dietary counseling and smoking cessation campaigns. Additionally, it is our opinion that together with the reproductive healthcare services that are provided in Jordan, everyone should have free or affordable access to genetic counseling to minimize miscarriages due to genetic disorders.

## 5. Conclusions

It is concluded that about one-fifth of the participating women had a history of a spontaneous miscarriage in their last pregnancy, with active smoking during pregnancy identified as a strong and preventable risk factor. Other risk factors were that the mother had more than four children, had a miscarriage-prone family, experienced problems in reproduction, received medical assistance for conception, and flew while pregnant. The study’s results hold implications for public health efforts aimed at reducing the incidence of spontaneous miscarriages in Jordan and provide valuable insights for guiding interventions and policy development in this area.

## Figures and Tables

**Table 1:** Sociodemographic characteristics, gynecologic, and obstetric history of participants (N = 704).

Characteristics					
		Overall Sample	No Miscarriage History Frequency (%)	Miscarriage History Frequency (%)	*p*-Value
Age	<22 years	26 (3.7)	23 (3.3)	3 (0.4)	0.076
	22–34 years	326 (46.3)	255 (36.2)	71 (10.1)	
	35–44 years	270 (38.4)	228 (32.4)	42 (6.0)	
	More than 44 years	82 (11.6)	72 (10.2)	10 (1.4)	
Residency	North Jordan	366 (52.0)	299 (42.5)	67 (9.5)	0.105
	Central Jordan	284 (40.3)	229 (32.5)	55 (7.8)	
	South Jordan	54 (7.7)	50 (7.1)	4 (0.6)	
Educational level	Secondary education or less	148 (21.0)	110 (15.6)	38 (5.4)	0.035 *
	College degree	138 (19.6)	115 (16.3)	23 (3.3)	
	Bachelor’s degree	315 (44.7)	263 (37.4)	52 (7.4)	
	Postgraduate degree	103 (14.6)	90 (12.8)	13 (1.8)	
Employment	Employed	322 (45.7)	271 (38.5)	51 (7.2)	0.191
	Unemployed	382 (54.3)	307 (43.6)	75 (10.7)	
Family income	JOD <500	343 (48.7)	270 (38.4)	73 (10.4)	0.064
	JOD 500–999	203 (28.8)	171 (24.3)	32 (4.5)	
	JOD 1000–1500	82 (11.6)	68 (9.7)	14 (2.0)	
	JOD >1500	76 (10.8)	69 (9.8)	7 (1.0)	
Body mass index (*n* = 694)	Underweight	17 (2.4)	16 (2.3)	1 (0.1)	0.404
	Normal weight	269 (38.8)	225 (32.4)	44 (6.3)	
	Overweight	262 (37.8)	213 (30.7)	49 (7.1)	
	Obese	146 (21.0)	116 (16.7)	30 (4.3)	
Smoking status	Smoker	150 (21.3)	114 (16.2)	36 (5.1)	0.028 *
	Non-smoker	554 (78.7)	464 (65.9)	90 (12.8)	
Husband smoking status	Smoker	405 (57.5)	325 (46.2)	80 (11.4)	0.135
	Non-smoker	299 (42.5)	253 (35.9)	46 (6.5)	
	Gynecologic and obstetric history				
Age at first menstruation	<11 years old	37 (5.3)	30 (4.3)	7 (1.0)	0.932
	11–13 years old	368 (52.3)	304 (43.2)	64 (9.1)	
	>13 years old	299 (42.5)	244 (34.7)	55 (7.8)	
Duration of flow	2 days or less	66 (9.4)	54 (7.7)	12 (1.7)	0.406
	3–7 days	598 (84.9)	488 (69.3)	110 (15.6)	
	8 days or more	40 (5.7)	36 (5.1)	4 (0.6)	
Amount of blood flow per day	Little (<5 pads per day)	376 (53.4)	308 (43.8)	68 (9.7)	0.199
	Moderate (5–10 pads per day)	306 (43.5)	255 (36.2)	51 (7.2)	
	Heavy (>10 pads per day)	22 (3.1)	15 (2.1)	7 (1.0)	
Cycle length	<21 days	137 (19.5)	115 (16.3)	22 (3.1)	0.402
	21–35 days	488 (69.3)	402 (57.1)	86 (12.2)	
	>35 days	8 (1.1)	5 (0.7)	3 (0.4)	
	Irregular	71 (10.1)	56 (8.0)	15 (2.1)	
Having gynecological disorder	Yes	97 (13.8)	72 (10.2)	25 (3.6)	0.029 *
	No	607 (86.2)	506 (71.9)	101 (14.3)	
Having fertility problems	Yes	123 (17.5)	78 (11.1)	45 (6.4)	<0.001 *
	No	581 (82.5)	500 (71.0)	81 (11.5)	
Husband fertility problems	Yes	59 (8.4)	35 (5.0)	24 (3.4)	<0.001 *
	No	645 (91.6)	543 (77.1)	102 (14.5)	
Number of children	None	111 (15.8)	88 (12.5)	23 (3.3)	<0.001 *
	1 child	316 (44.9)	267 (37.9)	49 (7.0)	
	2–3 children	186 (26.4)	163 (23.2)	23 (3.3)	
	4 or more	91 (12.9)	60 (8.5)	31 (4.4)	
Status of last pregnancy	Natural conception	676 (96.0)	564 (80.1)	112 (15.9)	<0.001 *
	Medically assisted conception	28 (4.0)	14 (2.0)	14 (2.0)	
Family history of abortion	Yes	158 (22.4)	118 (16.8)	40 (5.7)	0.006 *
	No	546 (77.6)	460 (65.3)	86 (12.2)	

* *p*-value < 0.05 are considered significant.

**Table 2:** Logistic regression analysis of risk factors of miscarriage.

Variables		OR	95% CI	*p* Value
Smoking		1.78	1.09–2.89	0.021 *
Number of children who resulted from previous pregnancies	None (reference)			0.001 *
	One child	0.95	0.51–1.73	0.861
	2–3 children	0.65	0.32–1.31	0.225
	4 or more	2.51	1.23–5.12	0.011 *
Having fertility problems		2.48	1.44–4.27	0.001 *
The last pregnancy was achieved naturally (reference) or after medical assistance?		2.65	1.01–6.96	0.048 *
Family history of miscarriage		1.89	1.18–3.04	0.008 *
Air travel		2.71	1.09–6.73	0.032 *

OR = odds ratio; CI = confidence interval; * *p*-value < 0.05 is considered significant.

## Data Availability

The datasets generated during and/or analyzed during the current study are available from the corresponding author on reasonable request.
